# Rationale, application and clinical qualification for NT-proBNP as a surrogate end point in pivotal clinical trials in patients with AL amyloidosis

**DOI:** 10.1038/leu.2016.191

**Published:** 2016-08-02

**Authors:** G Merlini, I Lousada, Y Ando, A Dispenzieri, M A Gertz, M Grogan, M S Maurer, V Sanchorawala, A Wechalekar, G Palladini, R L Comenzo

**Affiliations:** 1 Amyloid Research and Treatment Center, Department of Molecular Medicine, University of Pavia, IRCCS Policlinico San Matteo, Pavia, Italy; 2 Amyloidosis Research Consortium, Inc., Boston, MA, USA; 3 Department of Neurology, Graduate School of Medical Sciences, Kumamoto University, Kumamoto, Japan; 4 Mayo Clinic College of Medicine, Rochester, MN, USA; 5 Clinical Cardiovascular Research Laboratory for the Elderly, Columbia University Medical Center, New York, NY, USA; 6 Amyloidosis Center, Boston University School of Medicine and Medical Center, Boston, MA, USA; 7 Reader in Medicine and Haematology, National Amyloidosis Centre, University College London, London, UK; 8 The John C. Davis Myeloma and Amyloid Program, Tufts Medical Center, Boston, MA, USA

## Abstract

Amyloid light-chain (LC) amyloidosis (AL amyloidosis) is a rare and fatal disease for which there are no approved therapies. In patients with AL amyloidosis, LC aggregates progressively accumulate in organs, resulting in organ failure that is particularly lethal when the heart is involved. A significant obstacle in the development of treatments for patients with AL amyloidosis, as well as for those with any disease that is rare, severe and heterogeneous, has been satisfying traditional clinical trial end points (for example, overall survival or progression-free survival). It is for this reason that many organizations, including the United States Food and Drug Administration through its Safety and Innovation Act Accelerated Approval pathway, have recognized the need for biomarkers as surrogate end points. The international AL amyloidosis expert community is in agreement that the N-terminal fragment of the pro-brain natriuretic peptide (NT-proBNP) is analytically validated and clinically qualified as a biomarker for use as a surrogate end point for survival in patients with AL amyloidosis. Underlying this consensus is the demonstration that NT-proBNP is an indicator of cardiac response in all interventional studies in which it has been assessed, despite differences in patient population, treatment type and treatment schedule. Furthermore, NT-proBNP expression is directly modulated by amyloidogenic LC-elicited signal transduction pathways in cardiomyocytes. The use of NT-proBNP will greatly facilitate the development of targeted therapies for AL amyloidosis. Here, we review the data supporting the use of NT-proBNP, a biomarker that is analytically validated, clinically qualified, directly modulated by LC and universally accepted by AL amyloidosis specialists, as a surrogate end point for survival.

## Introduction

Amyloid light-chain (LC) amyloidosis (AL amyloidosis), a rare, progressive and fatal disease, is the most common form of systemic amyloidosis,^1, 2^ and it affects an estimated 8–12 per 1 000 000 persons annually.^3, 4^ Systemic amyloidosis is characterized by the accumulation of abnormal, misfolded protein (amyloid) in various tissue and organs that produce patient-specific clinical manifestations depending on the organ impacted. Progressive amyloid deposition and proteotoxic effects of amyloid proteins lead to organ failure, which is especially catastrophic when the heart is affected, and is the primary cause of death.^1, 5^ As many as 70% of patients with AL amyloidosis have predominantly cardiac amyloid deposition.^2, 5^ The prevalence of all types of cardiac amyloidosis is assumed to be underestimated because of missed diagnoses, given that the symptoms of cardiac amyloidosis often mimic those of other far more common conditions.^6, 7, 8^

There are no approved therapies for AL amyloidosis. That said, frequently used treatment options, such as high-dose chemotherapy in combination with autologous stem cell transplantation, alkylating agents, steroids, proteasome inhibitors and/or immunomodulatory drugs, reduce production of the amyloid-forming precursor protein, immunoglobulin LC, by targeting plasma cells.^1, 2, 9, 10^ Overall, plasma cell-directed therapies, also called source therapies, can induce a reduction in the concentration of the toxic LC by ⩾50% (partial response or better) in ~60% of patients, and this translates into cardiac or renal response, or both, in ~20–35% of patients. Cardiac response translates into substantial survival benefit.^2, 10, 11^

## Biomarkers as surrogate end points for rare diseases

A significant barrier to therapeutic development is posed by all diseases that are serious, heterogeneous and rare; for such diseases, traditional trials with clinical end points are difficult, and sometimes impossible, to conduct.^12^ It is for this reason that the use of biomarkers as surrogate end points was proposed by the United States Food and Drug Administration (FDA) with its Safety and Innovation Act Accelerated Approval pathway^12, 13^ to hasten the approval of new treatments. Furthermore, this position was recently promoted by the FDA: ‘The path to development of promising therapeutics can be enabled by the availability of biomarkers that are analytically validated and clinically qualified for a specific context of use'.^14^

The international AL amyloidosis expert community is in agreement that the N-terminal fragment of the pro-brain natriuretic peptide (NT-proBNP) is analytically validated and clinically qualified as a biomarker for use as a surrogate end point for survival in patients with AL amyloidosis. This consensus is supported by the consistent demonstration of the predictive value of NT-proBNP in all intervention trials to date, which we review here, and is especially striking in light of differences in study design, treatment regimens or combinations of treatment regimens, treatment class, patient population and geographic location. The reliability of NT-proBNP as a biomarker results from a direct intracellular pathway connecting the amyloid-forming precursor protein, immunoglobulin LC, to the induction of kinases known to promote *BNP* expression, and it makes the use of NT-proBNP, a robust surrogate for survival unique from other applications (for example, heart failure) in which inconsistencies have justifiably warranted apprehension. This rare and fatal disease should be considered a priority for expediting new treatments. In line with the Accelerated Approval pathway,^13^ a surrogate end point must be reasonably likely to predict a treatment's clinical benefit so that it will be considered validated and acceptable by regulatory authorities. In accordance with the FDA's criteria for Accelerated Approval, the following qualifying criteria for using a surrogate end point are met for AL amyloidosis:

‘…treats a serious condition…'—this is relevant for the substantial morbidity and mortality associated with AL amyloidosis. ‘…demonstrates an effect on a surrogate endpoint that is reasonably likely to predict clinical benefit or on a clinical endpoint that can be measured earlier than irreversible morbidity or mortality (IMM) that is reasonably likely to predict an effect on IMM…'—hematologic response, a commonly used efficacy measure for AL amyloidosis patients, may not capture the specific impact on heart dysfunction, which causes death. Consistent evidence from several independent clinical trials uniformly supports that NT-proBNP not only reasonably, but highly likely predicts clinical benefit to the heart. The use of a survival end point requires unacceptably extended trial durations for initial approval but could be confirmatory.

Without acceptance of surrogate end points for survival, a primary obstacle of AL amyloidosis clinical trials is their long duration and large study size, necessary because of the need for end points to demonstrate efficacy (foe example, overall survival and organ failure) with appropriate statistical power. Furthermore, the availability of several effective lines of plasma cell-directed therapy makes it difficult to evaluate the impact of a specific treatment on overall survival, and significant morbidity caused by organ dysfunction makes it difficult to define progression-free survival. Acceptance by the regulatory authorities of this important surrogate will accelerate drug development by facilitating more rapid clinical development of molecules and will standardize study designs evaluated in clinical trials (for example, treatment dose and duration, patient inclusion criteria and interpretation of multiple treatments). This will lead to more drug-industry investment into the study of AL amyloidosis. At present, ‘source therapies' are being used off-label, and few manufacturers are pursuing regulatory approval because of the long development time, difficult trial design and significant investment required for such a small population.

Although controlling the source of the amyloid precursor protein and factoring hematologic response into efficacy measures is important, they are of limited long-term value to patients in the absence of organ improvement.^15^ For this reason, using NT-proBNP as a surrogate end point for pivotal clinical trials in patients with AL amyloidosis will advance therapeutic treatments that save lives. Here, we present the data and rationale for the use of NT-proBNP, a biomarker that is analytically validated, clinically qualified, directly modulated by LC-elicited signal transduction pathways in cardiomyocytes and accepted by AL amyloidosis specialists as a surrogate end point for survival.

## Cardiac amyloidosis

In AL amyloidosis, misfolded LCs predominantly affect the heart (~70% of patients), kidneys (70% of patients) or both, but can also affect the liver, gastrointestinal tract, soft tissue, and peripheral and autonomic nervous systems (<20% patients each).^1, 10, 16^ The extent of cardiomyopathy is the most important determinant of outcome in patients with systemic amyloidosis.^2, 5, 17^ As mentioned, no therapies have been specifically approved for the treatment of patients with AL amyloidosis, and optimal treatment regimens remain undefined.^9, 18^

Extracellular amyloid deposits in the heart create anatomic restrictions that contribute to cardiac dysfunction.^19^ Amyloid-forming LCs also cause rapid and direct myocardial toxicity,^20, 21^ eliciting oxidative damage,^22, 23^ possibly through interactions with mitochondrial proteins.^24^ Amyloid LCs purified from patients with amyloid cardiomyopathy induce p38 mitogen-activated protein kinase (MAPK) signaling,^25^ resulting in oxidative stress, impaired excitation-contraction coupling^22, 26^ and, eventually, cardiomyocyte death.^25, 26^

Mayo Clinic investigators recently reported that hematologic response alone is not necessarily adequate to result in clinical benefit, and that early organ response predicts improved overall survival after successful therapy in AL.^15^ The development of therapies designed to decrease levels of circulating precursor, clear toxic cardiac aggregates and accelerate the clearance of amyloid deposits addresses a critical unmet need to reverse or attenuate cardiomyopathy in patients with AL amyloidosis. The use of NT-proBNP will facilitate the emergence of such novel therapies.

## Biological significance of nt-proBNP

NT-proBNP has emerged as an analytically validated, gold standard biomarker for the determination of cardiovascular risk and disease.^27^ In some patients, NT-proBNP assessment may facilitate a better diagnosis than physician review of patient history and other laboratory findings.^28, 29^ Moreover, the response of NT-proBNP after intervention is often used in clinical trials to assess outcome and cardiac progression. NT-proBNP has been clinically qualified to a great extent in AL amyloidosis, and AL amyloidosis specialists agree that it predicts cardiac response and improved clinical outcome after intervention. Thus, NT-proBNP, in contrast to other cardiac markers (for example, cardiac troponins), is the focus of this article.

BNP is a myocyte-secreted hormone that maintains fluid homeostasis in the body through natriuretic, diuretic and vasodilatory effects. BNP is secreted in response to ventricle distension and stretching caused by volume expansion and pressure overload.^30, 31^ Cardiomyocytes synthesize a pre-propeptide (preproBNP; 134 amino acids), which is cleaved to the propeptide (proBNP; 108 amino acids). After secretion, proBNP is cleaved to the active hormone BNP (amino acids 77–108) and the biologically inactive peptide NT-proBNP (amino acids 1–76).^31, 32^ Notably for AL amyloidosis, MAPK signaling mediates BNP transcription,^33, 34, 35^ supporting a direct connection between LC cardiotoxic effects with induced MAPK signaling and BNP levels. This direct modulation of BNP synthesis by cardiotoxic LC amyloid precursor is distinct from the processes proposed to elicit BNP secretion in other cardiovascular diseases and makes BNP levels directly reflective of the LC-induced cardiac pathology in AL amyloidosis. Ventricular dysfunction is indicated by increased serum BNP and NT-proBNP, which are secreted at a 1:1 equimolar basis.^32^ In the circulatory system, NT-proBNP has a longer half-life than BNP (2 h vs 22 min) and is subject to renal clearance.^30, 32^

Measurement of NT-proBNP is a robust, standardized laboratory test than can be easily and rapidly performed worldwide (1 h using electrochemiluminescence immunoassay).^36^ European Cardiology Society guidelines for the diagnosis and treatment of acute and chronic heart failure include measurement of natriuretic peptides among the essential initial investigations, reporting that a normal natriuretic peptide level in an untreated patient virtually excludes significant cardiac disease, making echocardiography unnecessary.^29^ Furthermore, in their recent document defining cardiovascular end points for clinical trials, the American College of Cardiology/American Heart Association included NT-proBNP/BNP as laboratory evidence supporting the diagnosis of worsening heart failure.^37^

Circulating NT-proBNP levels increase with patient age and renal insufficiency, are higher in women than men and are generally lower in obese patients because of the presence of NT-proBNP receptors on adipocytes and the production of neprilysin in adipose tissue. On the basis of the biological variability, resulting in a critical difference/reference change value of ~25% of circulating NT-proBNP,^38, 39^ clinically meaningful response has been defined as a >30% decrease of NT-proBNP, a reduction of ⩾300 ng/l (35.4 pmol/l) in patients with ⩾650 ng/l (76.7 pmol/l) baseline NT-proBNP.^11, 40^

No clear superiority of BNP compared with NT-proBNP has been established in the biomarker field; however, studies performed in patients with AL amyloidosis have primarily used NT-proBNP. This might be because NT-proBNP has a longer half-life, is more sensitive and more stable (for example, can be performed on frozen serum) than BNP, which requires stricter pre-analytical requirements.

Measuring circulating NT-proBNP is more rapid, more reproducible, more affordable and more accessible than evaluations that require specialized equipment, procedures (for example, echocardiography) and trained personnel,^29^ and may allow for the detection of meaningful cardiovascular responses in advance of morphologic changes found on echocardiography.^41^ NT-proBNP is the most sensitive marker of cardiac involvement in AL amyloidosis; it has 100% sensitivity for detecting cardiac involvement estimated by clinical signs, electrocardiography and echocardiography using the validated cutoff of 332 ng/l (0.33 mg/ml or 39.2 pmol/l).^42^

## Application of cardiac biomarkers to patients with AL amyloidosis

Several studies demonstrate that cardiac biomarkers, particularly NT-proBNP, are powerful predictors of prognosis in AL amyloidosis.^42, 43^ Current staging systems for this disease are based on serum levels of NT-proBNP, cardiac troponin T and the concentration of circulating amyloidogenic-free LCs.^44^ In addition to the relevance of NT-proBNP in prognostic stratification at baseline, changes in NT-proBNP concentration after therapy predict clinical outcomes for patients. Specifically, NT-proBNP response or progression predicts attenuation or progression of cardiac dysfunction, with significant impact on survival.^11^

## Baseline NT-proBNP as a prognostic factor

**Figure 1:**
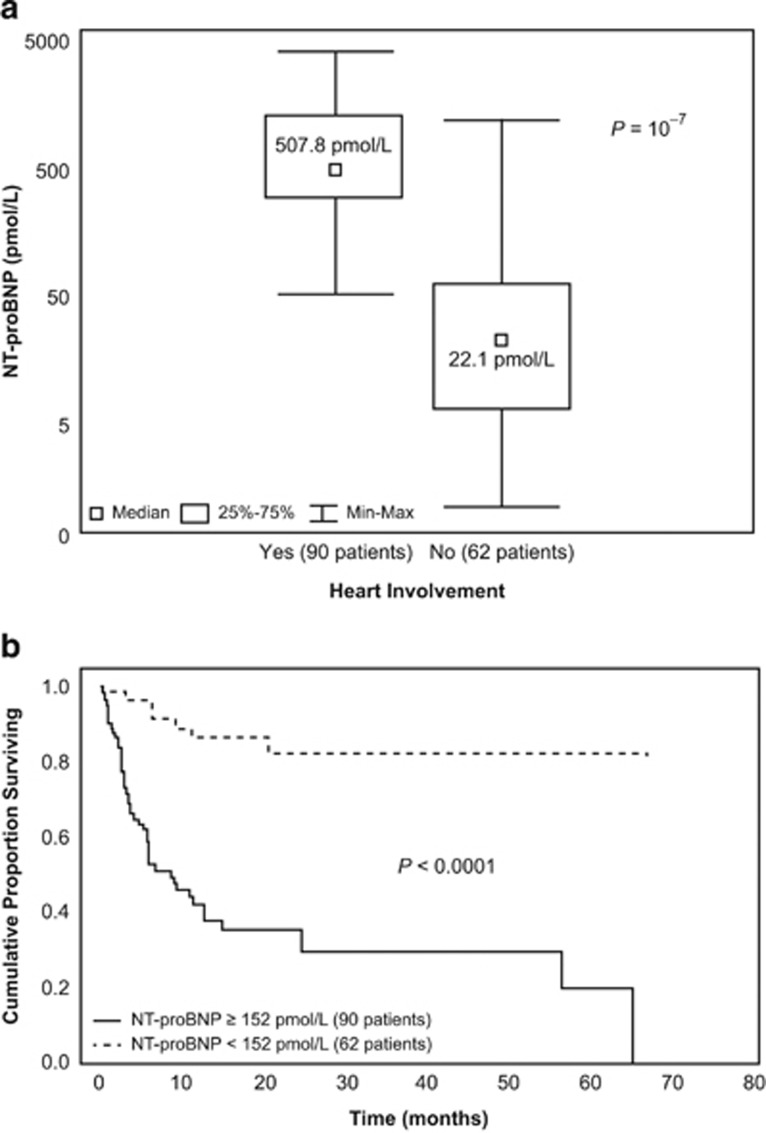
NT-proBNP levels indicate cardiac involvement (**a**) and predict overall survival (**b**) in Palladini *et al.*^42^ Adapted with permission from Palladini *et al.*^42^ Max, maximum; Min, minimum.

Nine studies^42, 45, 46, 47, 48, 49, 50, 51, 52^ demonstrate that baseline NT-proBNP level predicts clinical outcome in patients with newly diagnosed AL amyloidosis. Palladini *et al.*^42^ first reported an association between baseline NT-proBNP levels with cardiac involvement and survival in 152 patients with AL amyloidosis (Figure 1). In separate publications, Dispenzieri *et al.*^53^ demonstrated that baseline NT-proBNP levels predicted survival in 242 patients with newly diagnosed amyloidosis and in 98 patients before they underwent autologous stem cell transplantation (ASCT).^51^ Kristen *et al.*^50^ demonstrated that baseline NT-proBNP level was an independent predictor of survival in 163 patients with AL amyloidosis. In an analysis of 1998 patients seen over a 30-year period at the Mayo Clinic compared with 313 contemporary patients, Kumar *et al.*^45^ reported that NT-proBNP levels consistently identified patients at risk for early death despite improvements in survival during the same period. Wechalekar *et al.*^52^ demonstrated that presenting NT-proBNP correlated with survival in a group of 346 patients with newly diagnosed AL amyloidosis who subsequently were treated with hematologic therapies. Kristen *et al.*^46^ showed that baseline NT-proBNP level was a univariate predictor of survival in 185 patients with AL amyloidosis. Banypersad *et al.*^47^ demonstrated, in 100 patients with AL amyloidosis, that cardiac disease revealed by nuclear magnetic resonance imaging correlated with NT-proBNP levels. Kristen *et al.*^48^ confirmed that risk stratification associated with both NT-proBNP and cardiac troponin T predicted 1-year mortality in 125 patients with AL amyloidosis. These 9 independent studies in 3722 treatment-naive patients show that NT-proBNP responses consistently reflect changes in cardiac function and predict survival in patients with AL amyloidosis.

## NT-proBNP response after intervention predicts clinical outcome

In the context of interventional therapy, 5 large independent studies^11, 49, 54, 55, 56^ have established that the NT-proBNP response, defined as a decrease in NT-proBNP of >30% and >300 ng/l (35.4 pmol/l) in evaluable patients (those whose baseline NT-proBNP levels were ⩾650 ng/l; 76.7 pmol/l), predicts clinical outcome and survival. NT-proBNP is a survival marker independent of therapy type, treatment class, or regimen; these studies represent patients treated with 9 different combinations of therapies and 3 different individual therapies, which include chemotherapies and ASCT as well as steroids, immunomodulatory drugs, proteasome inhibitors, and alkylating agents (Table 1).

**Figure 2:**
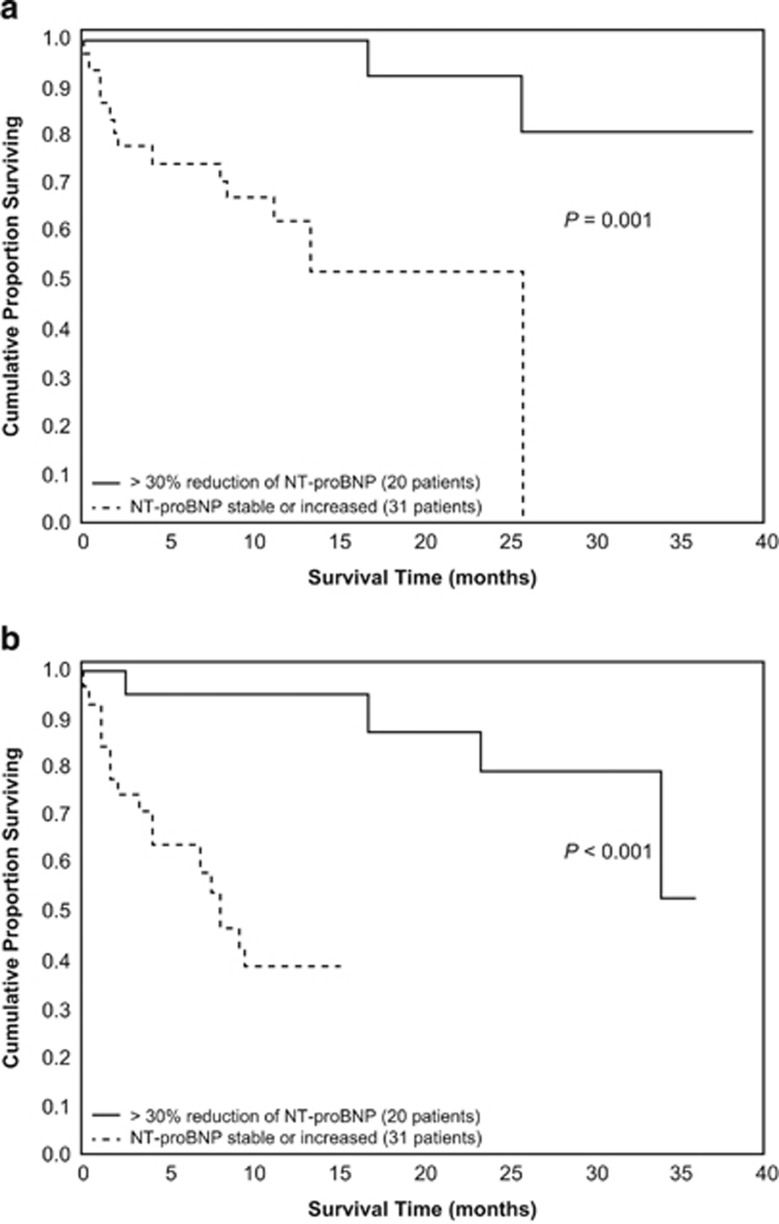
Overall (**a**) and progression-free (**b**) survival with respect to NT-proBNP response in Palladini *et al.*^54^ Adapted with permission from Palladini *et al.*^54^

**Figure 3:**
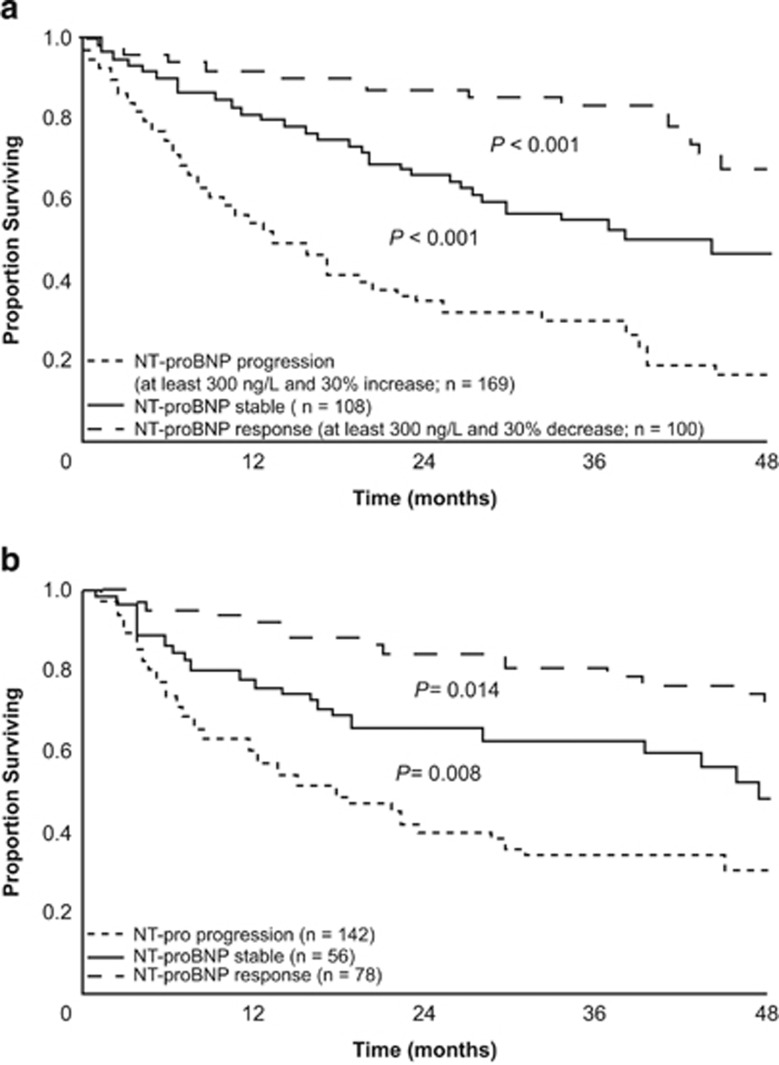
Survival vs NT-proBNP response and progression in testing (**a**) and validation (**b**) groups in Palladini *et al.*^11^ Adapted with permission from Palladini *et al.*^11^

Palladini *et al.*^54^ documented, in 51 patients with cardiac AL amyloidosis treated with melphalan plus dexamethasone (MDex), thalidomide plus dexamethasone (TDex), dexamethasone (Dex), melphalan plus prednisone (MP) or thalidomide (T), that achievement of NT-proBNP response predicted both overall survival and progression-free survival (Figure 2). Subsequently, Kastritis *et al.*^55^ showed that the post-treatment NT-proBNP (and BNP) response independently predicted survival in 94 patients with AL amyloidosis treated with bortezomib (Bor) or Bor plus dexamethasone (BDex). Palladini *et al.*^49^ demonstrated that post-treatment NT-proBNP response independently predicted patient survival in a study of 113 patients with AL amyloidosis treated primarily with MDex, Dex, cyclophosphamide plus thalidomide and dexamethasone (CyTDex) or ASCT. Kastritis *et al.*^56^ reported that NT-proBNP response predicted survival in 85 patients with AL amyloidosis treated with BDex or with L-based or risk-adapted BDex. On the basis of these results, in 2012 the International Society of Amyloidosis established and validated NT-proBNP response as an indicator of organ response and as a surrogate marker of survival in AL amyloidosis. NT-proBNP response predicted a significant survival benefit both in testing (*n*=816) and in validation (*n*=374) populations treated primarily with MDex, T-based, lenalidomide (L)-based, Bor-based, Dex, MP or ASCT treatments (Figure 3).^11^ Notably, this international study in 1190 patients failed to show any survival benefit of ⩾2-mm reduction in the thickness of the cardiac interventricular septum, which had been used as a criterion for cardiac response.^56^

**Figure 4:**
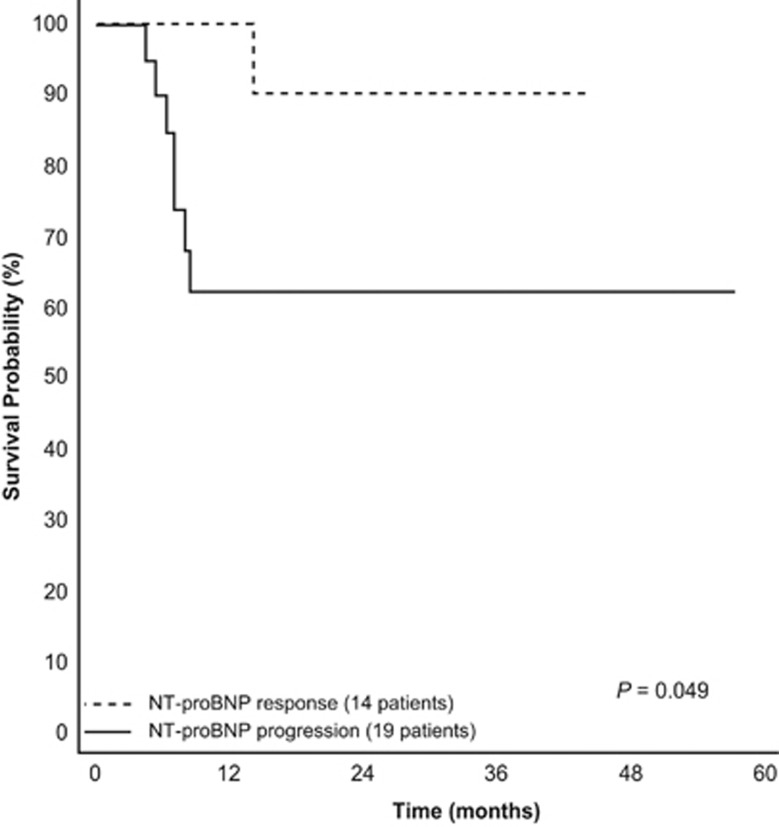
Survival according to NT-proBNP response in an ongoing phase 3 trial comparing melphalan-dexamethasone with melphalan-bortezomib-dexamethasone (NCT01277016).

Overall, these 5 independent studies^11, 48, 54, 55, 56^ in 1482 patients after interventional treatment show that NT-proBNP responses consistently reflect changes in cardiac function and predict survival in patients with AL amyloidosis. However, these studies were retrospective. Prospectively, the response criteria are being used in an ongoing phase 3 study comparing MDex with MBDex (NCT01277016). Although the study is not yet complete and the number of evaluable patients is limited, the preliminary outcome analysis indicates that NT-proBNP response translates into a significant survival benefit (Figure 4).

AL amyloidosis is an exceedingly rare disease. To put it in context, AL amyloidosis with cardiac involvement is diagnosed in ~3000 new patients each year in the US^57^ The post-intervention validation sample set of 5 studies^11, 48, 54, 55, 56^ represents roughly 49% of all new diagnoses per year. Even in multiple myeloma, ~14 000 patients would be needed to reach the average number of new diagnoses this year in the US for a similar validation set.

In patients with both renal and cardiac involvement, reductions in glomerular filtration rate can influence NT-proBNP levels. In a study of 248 such patients, stratification by glomerular filtration rate (⩾60 ml/min/1.73 m^2^; <60 and ⩾15 ml/min/1.73 m^2^; or <15 ml/min/1.73 m^2^) revealed that though decreasing glomerular filtration rate required a higher threshold for detecting heart involvement and predicting survival, NT-proBNP predicted survival in patients with glomerular filtration rates⩾15 ml/min/1.73 m^2^.^58^

Although several cardiac biomarkers and imaging techniques have been reported to have a prognostic value, none have been validated as surrogate markers of cardiac response and clinical outcome in patients with AL amyloidosis. There is no clear correlation between reductions in NT-proBNP with improvements in cardiac anatomy,^54^ which may reflect potentially differing time courses in detectable changes in cardiac structure compared with hormone secretion and/or a distinct role served by LC in the modulation of BNP expression through a direct cytotoxic mechanism. More refined echocardiographic assessment, such as tissue Doppler imaging and strain rate imaging, may ultimately add to the prognostic discrimination based on biomarkers at baseline, but this application requires systematic study in patients with cardiac amyloidosis, and there is no useful precedent for monitoring response to therapy (reviewed in studies by Gertz *et al.*^59^ and Falk *et al.*^60^). Similarly, recent studies^61^ have proposed cardiac magnetic resonance imaging as an additional prognostic tool in cardiac AL amyloidosis, but no study has yet explored cardiac magnetic resonance imaging in response assessment. Notable additional strengths of NT-proBNP assessment are its robust standardization, reproducibility, ease, speed and low-cost compared with more expensive and complex imaging techniques that are frequently operator dependent.

Although other biomarkers (including high-sensitivity troponin^62^ and novel biomarkers such as ST2,^63^ GDF-15,^64^ mid-regional pro-adrenomedullin^65^ and osteoprotegerin^66^) measured at diagnosis are prognostic factors in patients with AL amyloidosis, they have not yet been predictive of survival based on response after interventional treatment. More evidence is needed to establish their potential value. Only NT-proBNP response after several different classes of treatment indicated patient outcome and could be used as a marker of response. Increased cardiac troponin indicated disease progression and poor survival in patients with AL amyloidosis, but the role for troponin in the definition of response remains less established.^11, 49, 62, 66, 67^ Finally, NT-proBNP should continue to be evaluated for use in assessing cardiac response in patients with both mutated and wild-type cardiac transthyretin amyloidosis.

## Summary and conclusion

**Figure 5:**
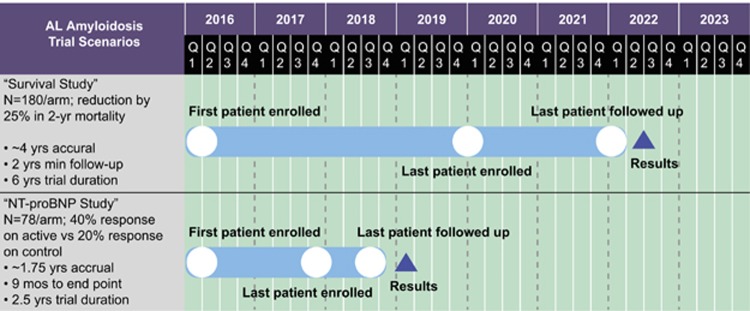
Estimated time required to complete a trial in patients with AL cardiomyopathy using overall survival rather than NT-proBNP response as the end point.

NT-proBNP is a unique biomarker of cardiac amyloid involvement in patients with AL amyloidosis. It is of fundamental importance for establishing diagnosis, prognosis, and response to therapy in AL cardiomyopathy. This formidable clinical impact derives from the direct regulation of NT-proBNP levels by pathologic processes downstream of cardiac LC signaling (that is, MAPK activation), making NT-proBNP an unambiguous marker of amyloid cardiac disease. The use of NT-proBNP as a surrogate efficacy end point for AL amyloidosis trials using current, validated definitions of response is not controversial. NT-proBNP level should be widely adopted to evaluate the effectiveness of new treatments targeting cardiac dysfunction in patients with AL amyloidosis as an accepted primary outcome. Experts agree that NT-proBNP is the only surrogate end point for survival after treatment, and lowering NT-proBNP and achieving NT-proBNP response are the ultimate treatment objectives. Overall survival as an end point requires a larger study population and a longer assessment period; a trial using NT-proBNP could be half as long (Figure 5). The use of this end point is particularly important to minimize treatment-related toxicity and mortality and to conduct systematic clinical research more rapidly.

A recent meta-analysis^68^ of the impact of biomarker-based strategies on oncology drug development from clinical trials that led to FDA approval supported the safety and improved efficacy outcomes in FDA-approved anticancer agents using biomarker-based tactics. This approach is now feasible and necessary in AL amyloidosis. Now is a pivotal moment for many novel drugs in the pipeline that promise to address unmet needs in this rapidly evolving, but curable, disease. We owe it to our patients to rapidly assess the efficacy of new drugs. The data presented here establish that the NT-proBNP response, defined as a decrease in NT-proBNP of >30% and >300 ng/l (35.4 pmol/l), predicts clinical outcome and survival and is a validated and qualified surrogate marker of efficacy for interventions for patients with AL amyloidosis.

## Figures and Tables

**Table 1:** Summary of clinical trials demonstrating that NT-proBNP response after intervention predicts clinical outcome

*Study*	*Patient population (median age)*	*Cardiac involvement, %*^a^	*No. of subjects (male, %)*	*Treatment regimen*	*Median survival*	
					*NT-proBNP responders*^b^	*NT-proBNP non-responders*
Palladini *et al.*^54^	No previous treatments (63 years)	100	51 (53)	MDex, TDex, Dex, MP or T	>80% at 40 months	~13 months
Kastritis *et al.*^55^	Newly diagnosed and previously treated (62 years)	62	94 (52)	Bor, BDex	>80% at 36 months	~12 months
Palladini *et al.*^49^	Newly diagnosed (64 years)	37	171 (58)	MDex, CyTDex, Dex, ASCT, ‘other'	>80% at 60 months	8 months
Palladini *et al.*^11^	Newly diagnosed (63 years)	69	Testing cohort 816 (60)	MDex, T-based, L-based, Bor-based, Dex, MP, ASCT, ‘other'	>65% at 48 months	~10 months
Palladini *et al.*^11^	Newly diagnosed (64 years)	84	Validation cohort 374 (60)	—	>75% at 48 months	~15 months
Kastritis *et al.*^56^	Newly diagnosed (57 years)	44	85 (57)	BDex, L-based, risk-adapted BDex	~45 months	~10 months

Abbreviations: ASCT, autologous stem cell transplantation; BDex, bortezomib plus dexamethasone; Bor, bortezomib; CyTDex, cyclophosphamide plus thalidomide and dexamethasone; Dex, high-dose dexamethasone; L, lenalidomide; MDex, melphalan plus high-dose dexamethasone; MP, melphalan plus prednisone; NT-proBNP, N-terminal fragment of the pro-brain natriuretic peptide; T, thalidomide; TDex, thalidomide plus intermediate-dose dexamethasone.

a Cardiac involvement=percentage of patients with New York Heart Association class ⩾2.

b Median survival not reached.
